# Green and Sustainable Clay Ceramic Membrane Preparation and Application to Textile Wastewater Treatment for Color Removal

**DOI:** 10.3390/membranes15100292

**Published:** 2025-09-26

**Authors:** Jamila Bahrouni, Afef Attia, Fatima Zohra Elberrichi, Lasâad Dammak, Lassaad Baklouti, Mohamed-Ali Ben Aissa, Raja Ben Amar, Andre Deratani

**Affiliations:** 1 Research Unit “Advanced Technologies for Environment and Smart Cities”, Faculty of Sciences, University of Sfax, Sfax 3000, Tunisia; jamilabahrouni2020@gmail.com (J.B.); afef.attia@fss.usf.tn (A.A.); raja.benamar@fss.usf.tn (R.B.A.); 2 Institut de Chimie et des Matériaux Paris Est, ICMPE UMR-CNRS 7182-UPEC, Université Paris-Est Créteil, 2 rue Henri Dunant, 94320 Thiais, France; 3 Institut Européen des Membranes, IEM UMR-5635, CNRS, ENSCM, Université Montpellier, Place Eugène Bataillon, 34095 Montpellier, France; 4 Laboratoire Génie Civil et d′hydraulique, Université 8 Mai 1945, Guelma BP401, Algeria; 5 Laboratory of Applied Chemistry and Natural Substances Resources and Environment, Faculty of Sciences of Bizerte, University of Carthage, Tunis 7021, Tunisia; lassaad.baklouti@fsb.ucar.tn; 6 Department of Chemistry, College of Science, Qassim University, Buraydah 51452, Saudi Arabia

**Keywords:** sustainable ceramic membranes, kaolin, almond shells, lime, textile wastewater discoloration, indigo blue dye

## Abstract

Ceramic membrane technology plays an important role in water and wastewater treatment. Strategic sourcing of various natural mineral resources has contributed to developing low-cost ceramic membranes. The combination with calcination of inorganic and organic wastes from domestic and agricultural activities results in fully sustainable ceramic membrane materials. In this work, ceramic membranes were developed using 96% clay, 2% almond shells and 2% lime. Sintering at 900, 950, and 1000 °C enabled the production of membranes (MK-900, MK-950, and MK-1000) in a clean, simple, and cost-effective manner. The average pore diameter and porosity decreased slightly from 44 to 42 nm and from about 30% to 26% with increasing sintering temperature, while the flexural strength increased from 25 to 40 MPa. The pure water permeability was 68 and 59 L·m^−2^·h^−1^·bar^−1^ for MK-900 and MK-950, respectively. Effective color (as Indigo blue) removal of 78% and 92% was observed for MK-900 and MK-950, respectively. More than 90% of the initial permeability was recovered after three cycles of dye filtration using water backwashing at each stage, indicating good fouling resistance of the prepared membranes.

## 1. Introduction

The increasing population and demand for water, as well as climate change, represent major challenges for the sustainable management of this vital resource. Water scarcity and degradation of water quality constitute key issues in achieving human-related sustainable development goals, not only those specifically related to water and aquatic ecosystems, but also those related to food security, human health, the urban environment, and economic growth. In this context, water reuse is becoming increasingly necessary, as it can significantly reduce the demand for fresh water and contribute to more efficient water management strategies [1].

Among the various technologies available to meet the above objectives, membrane technology is one of the most attractive in the field of physical separation processes. Indeed, this technique offers high-performance pollutant removal with cost-effective and energy-efficient scalingand on-line integration [2,3]. The thermal treatment of kaolin based membrane is generally performed at temperatures ranging from 1000 to 1200 °C. In contrast, our study utilized lower temperatures, specifically between 900 and 1000 °C. Operating within this reduced temperature range helps decrease energy consumption while preserving the desired material properties, offering notable benefits in sustainability and cost-effectiveness. Improving the performance and functionality of membranes in terms of production and selectivity, for example, for the elimination of emerging pollutants (microplastics, organic pollutants, pesticides, etc.), requires the development of new materials [4,5,6]. New issues have also arisen in recent years, such as the long-term usability of membranes, as well as their reusability and recyclability. On the other hand, a number of different approaches to waste reutilization have been proposed, with the aim of addressing both economic and environmental concerns, such as manufacturing management and waste treatment, to achieve zero waste production [7,8].

Ceramic membranes are a class of membranes with many specific properties, such as high chemical and thermal stability, good mechanical strength, long lifespans, and a wide range of achievable microstructures, porosities, and geometries [9]. They have found applications in important fields where these properties are essential to ensure reproducibility and durability, such as water treatment [9,10], pharmaceutical and food industries [11,12], and gas separation [13]. Ceramic membranes are prepared from different materials, including alumina [14], titania [15], silica [16], and zirconia [17]. In most cases, these membranes are costly due to the expensive raw materials and high sintering temperatures required for their manufacture. As a result, there is great interest in new routes, particularly those involving low-cost raw materials and lower manufacturing temperatures [18]. Natural minerals as raw materials and food-wastes as organic pore-forming additives offer a good alternative to conventional materials thanks to their ability to be calcined at relatively low temperatures [19]. In recent years, considerable efforts have been made, for example, to find suitable pore-forming agents such as activated carbon [20], natural phosphates [21], dolomite [22], and powders derived from household and agricultural waste [23] in order to develop low-cost ceramic membranes with appropriate characteristics in terms of porosity and pore sizes. Interestingly, bio-based ceramic membranes have been successfully prepared from a wide variety of food by-products such as rice husk [24], sugarcane bagasse [25], banana peels [26], coconut husk and eggshells [27], and walnut husks [28].

Kaolinite, almond shell and lime were chosen in this study to prepare green, sustainable ceramic membranes. Kaolin is an attractive material for the manufacture of low-cost ceramic membranes due to its natural abundance and the good physical and chemical properties of sintered products [29]. The choice of almond shell, an agricultural waste product, and lime was mainly motivated by their low cost, renewable nature, ecological character, and availability in the local Tunisian environment. To our knowledge, these two additives have received little attention. A short study on kaolin-based microfiltration disks has been reported using almond shells as pore-forming agent without details on preparation and permeation properties [30,31]. Ultrafiltration (UF) is a cost-effective process; however, conventional UF membranes are not efficient at removing dye compounds due to their relatively large pore size compared to dye molecule size. On the other hand, lime up to 20% has been used in combination with chemicals to prepare kaolin-based tubular supports with an average pore size of 8 µm [32].

The aim of this study was to fabricate tubular ceramic ultrafiltration membranes using Algerian kaolinite clay, almond shells, and lime. To this end, the lime content was reduced compared to the previous study in order to decrease pore size, while the addition of almond shells was expected to achieve good porosity. After extrusion and sintering, the obtained membranes were analyzed for their microstructure, physical and chemical properties. using FT-IR, XRD, TGA, XPS, and scanning electron microscopy (SEM). The performance of the prepared membrane in textile wastewater treatment by removing color, COD, and turbidity was investigated.

This work contributes to the current research trend of developing low-cost methods for the preparation of ceramic membranes using inexpensive raw materials and valorizing waste materials such as almond shells, which could significantly reduce production costs and increase the accessibility of ceramic membranes for water treatment.

## 2. Materials and Methods

### 2.1. Chemicals

The raw materials used to prepare the tubular composite ceramic membranes are listed below:

− Natural kaolinite clay from the northwest of Algeria. The chemical composition analysis showed that Algerian clays are mainly composed of silica and alumina (Table 1), as generally reported for this kind of material in the literature [33].
− Almond shells. They were collected locally from agricultural and food waste. After washing and drying, they were ground into powder using an electric grinder and passed through a 33-mesh sieve.
− Lime. It was locally sourced and obtained by calcining limestone in a traditional kiln.

The reconstituted textile effluent containing indigo blue (IB, C_16_H_10_N_2_O_2_) dye was provided by SITEX, Ksar Hellal (Tunisia). All aqueous solutions were prepared with deionized water (18 MΩ·cm^−1^, Millipore Milli-Q, Burlington, MA, USA). The pH was adjusted using HCl (0.1 and 0.01 M) or NaOH (0.1 and 0.01 M).

### 2.2. Preparation of MK Membranes

The membranes were prepared from an aqueous slurry composed of kaolin clay powder blended with almond shells and lime. MK tubular membranes were produced using a locally manufactured extruder, then sintered at various temperatures (900 °C, 950 °C and 1000 °C) named MK-900, MK-950 and MK-1000. To prevent cracking and deformation, the sintered membranes were dried at a speed of 5 °C/min. A two-stage program was defined: the first at 300 °C for 3 h and the second for sintering at high temperatures for 3 h. The tubes obtained were cut to a length of 12 cm and polished with sandpaper to obtain a final inner/outer diameter of 5/7 mm.

### 2.3. Membrane Characterization

The particle size distribution (PSD) analysis of the powders was carried out using a Mastersizer 2000 instrument (Malvern Instruments, Malvern, Worcestershire, UK). The raw material and membranes obtained were characterized by infrared spectroscopic analysis (FTIR Spectrum 100, Perkin Elmer, Waltham, MA, USA) and X-ray diffraction (XRD) (D8 Advance diffractometer, Bruker, Billerica, MA, USA) with Cu_K_ radiation (λ = 1.5046 Å), scanning from 4 to 80 in 2θ. The membrane surface morphology was studied by scanning electron microscopy (SEM) using a Hitachi S4800 (Tokyo, Japan). The chemical stability of the synthesized membrane was determined by measuring weight loss after immersing the membrane in acidic (pH = 3) and alkaline (pH = 12) solutions for 24 h. The membranes were then washed with distilled water, dried at 105 °C, and the final weight recorded [34]. The mechanical strength was measured using a three-point bending test on a mechanical testing machine (model LRX, Lloyd Instruments, Fareham, UK). The samples, of 45 × 12 × 2 mm^3^, were placed on two supports spaced 30 mm, and the maximum breaking force was recorded in Newton.

The percentage shrinkage (*D*) during the sintering process was determined by measuring the diameter and thickness of the ceramic membrane before and after sintering and calculated using Equation (1) [35].

(1) $$D\left(\%\right)=\frac{{(D}_{i}-D_{f})}{D_{i}}\cdot 100$$

where *D_i_* is the initial diameter/thickness ratio before sintering and *D_f_* the final diameter/thickness after the sintering process.

Apparent porosity was determined by the immersion method, according to Archimede’s principle and using water as the fluid, as specified by the standard ASTM C 20 (2000) [36]. The membrane was dried at 105 °C for 12 h to obtain its dry mass (*M_d_*). It was then immersed in distilled water for 24 h to determine its wet mass (*M*_w_), after removing excess water with absorbent paper. Each measurement was repeated three times, and the porosity percentage of MK membranes (ε) was determined using Equation (2).

(2) $$\varepsilon (\%)=\frac{\left(M_{w}-M_{d}\right)}{M_{d}}$$

The average pore size was calculated using the extended Hagen-Poiseuille relationship (Equation (3)) [37]:

(3) $$d=2\cdot \sqrt{8\cdot J_{w}\cdot \delta \cdot \frac{\tau \cdot \Delta X}{\varepsilon \cdot \Delta P}}$$

where *d* is the pore diameter (m), *δ* the water viscosity (Pa·s), *J_w_* the water flux (m·s^−1^), *ε* (%) the membrane porosity, *τ* the tortuosity factor (2.5 for spherical particles), ∆*X* the membrane thickness (m) and ∆*P* the applied transmembrane pressure (Pa).

### 2.4. Filtration Performances of MK Membranes

**Figure 1:**
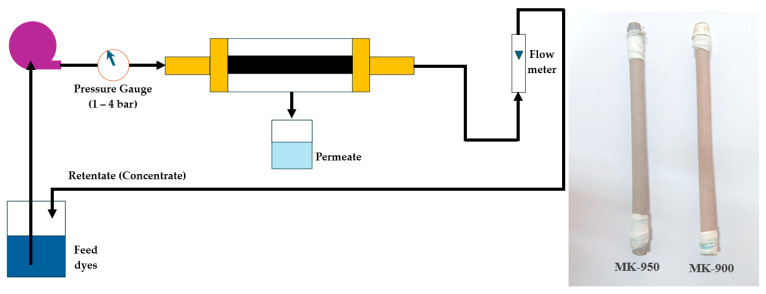
Schematic representation of the experimental system setup.

The filtration performance of MK membranes was determined in crossflow velocity mode. Figure 1 shows the experimental setup for the filtration studies, which comprises a membrane module with a tubular configuration to house the filters.

A 3 L tank was used for the feed, and the system was equipped with a positive displacement pump, a flow meter with flow control valves to measure and control the inflow rate, and a pressure gauge to monitor pressure changes in the system (Cole-Parmer, Vernon Hill, IL, USA). The feed solution was pumped into the system at a constant flow rate of 1.67 m·s^−1^. The retentate was recirculated to the feed tank, while the permeate was collected under atmospheric condition. The inlet pressure was indicated thanks to a pressure gauge [bar(g)], placed at the entrance of the membrane module.

As there is no gauge at the outlet, the outlet pressure was assumed to be slightly lower than the inlet pressure but close enough for approximation purposes due to the small size of the membrane module. This assumption was deemed acceptable for calculating permeate flux and membrane permeability. Permeate flux *J_v_* (L·m^−2^·h^−1^) was calculated using Equation (4), which takes into account the collected volume *V* (L), collection time (h), and effective membrane surface area *A* (m^2^).

(4) $$J_{v}=\frac{V}{A\cdot \Delta t}$$

#### 2.4.1. Water Permeability

Tests were carried out with deionized water at room temperature, varying the transmembrane pressure (Δ*P*). Membrane permeability *L_p_* (L·m^−2^·h^−1^·bar^−1^) was calculated using Darcy’s law [38]: (Equation (5)).

(5) $$J_{v}=L_{p}\cdot \Delta P\;with\;\Delta P=(\frac{P_{inlet}+P_{outlet}}{2})-P_{f}$$

where *J_v_* is the permeate water flux, Δ*P* the transmembrane pressure (bar), *P_inlet_* the inlet pressure (bar), *P_outlet_* the outlet pressure (bar) (*P_outlet_* approximatively equal to *P_inlet_*), and *P_f_* the filtrate pressure (*P_f_* = 0).

#### 2.4.2. Application to Dye Removal from Textile Wastewater

MK membranes were used to decolorize an aqueous solution representative of a textile industry effluent containing IB dye. The dye concentration in the solution matrix was 25 mg/L, which is commonly used in the textile industry, particularly in the production of denim clothing. Raw and treated wastewater were characterized by measurements of UV-Vis absorbance, pH, turbidity, and chemical oxygen demand (COD). Color intensity in feed and permeate solutions was determined using a spectrophotometer (UV 7205 Jenway, London, UK) at a wavelength of 610 nm, and turbidity using a turbidimeter (HACH, model 2100A, Ames, IA, USA) before and after the filtration experiment. The pH value was monitored during the experiment using a pH meter (SevenCompact pH meter S220, Mettler Toledo, Greifensee, Switzerland). Chemical oxygen demand (COD) was measured using the mercury-free LCK214 method. The textile effluent reconstituted in this study had a turbidity of 187 NTU, a COD of 147 mg·L^−1^, an absorbance of 1.132 at λ = 610 nm, and a pH of 8.5.

The retention value (*R*) for turbidity and COD was determined according to Equation (6):

(6) $$R(\%)=\frac{X_{f}-X_{p}}{X_{f}}\cdot 100$$

where *X_f_* and *X_p_* are the turbidity and COD values of the raw and treated solutions, respectively.

### 2.5. Membrane Fouling

The performance efficiency of MK membranes can be reduced by fouling, which decreases permeation flux and affects membrane selectivity. Membrane fouling resistance was determined by calculating flux recovery rate (FRR), total flux decline rate (*R_t_*), reversible flux decline rate (*R_r_*) and irreversible flux decline rate (*R_ir_*) using Equations (7)–(10), respectively [39]:

(7) $$FRR(\%)=\frac{J_{v_{2}}}{J_{v_{1}}}\cdot 100$$

(8) $$R_{t}(\%)=\frac{J_{v_{1}}-J_{v_{f}}}{J_{v_{1}}}\cdot 100$$

(9) $$R_{r}(\%)=\frac{J_{v_{2}}-J_{v_{f}}}{J_{v_{1}}}\cdot 100$$

(10) $$R_{ir}(\%)=\frac{J_{v_{1}}-J_{v_{2}}}{J_{v_{1}}}\cdot 100$$

*J*_*v*1_ is the pure water flux before filtration and *J_vf_* the permeate flux during the filtration step. The membrane was then regenerated by rinsing it for 20 min with deionized water, followed by treatment with a 2% NaOH solution at 80 °C. Deionized water was filtered through the membrane until a neutral pH was reached. Finally, the pure water flux (*J_v_*_2_) was determined for each cleaned membrane.

### 2.6. Membrane Regeneration

The membrane regeneration was first carried out by rinsing with water, followed by an acid–base treatment. This involved circulating a 2 wt.% sodium hydroxide (NaOH) solution at 80 °C for 30 min, then a 2 wt.% nitric acid (HNO_3_) solution at 60 °C for another 30 min. Finally, the membrane was rinsed with distilled water until neutral pH was achieved [40].

The cleaning protocol’s effectiveness was verified by measuring water permeability after regeneration. Permeability close to that of a new membrane is expected, with an acceptable reduction of about 10% after approximately fifty regeneration cycles.

## 3. Results

### 3.1. Characterization of the Raw Materials

#### 3.1.1. Particle Size Distribution

**Figure 2:**
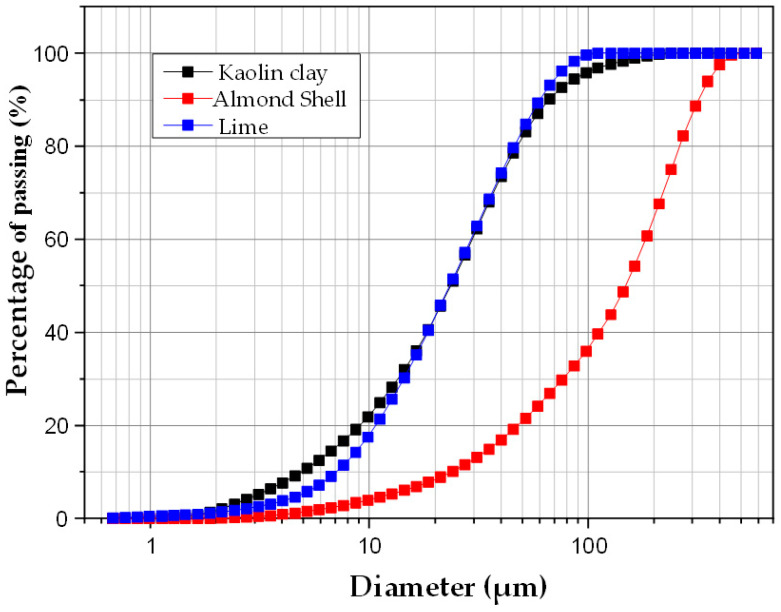
Particle size distribution after grinding and sieving of the raw materials used in this work.

Laser granulometric analysis of the raw materials, after grinding and sieving through a 100 μm mesh (kaolin and lime) and a 500 μm mesh (almond shell) prior to use in membrane fabrication, enabled the determination of average particle size and relative distribution. Figure 2 shows the particle size distributions of clay, lime and almond shells powders. As can be seen, lime and kaolin particles exhibited a similar distribution, with an average particle size of about 30 μm. In contrast, almond shell powder was composed of larger particles, with an average size of around 100 μm.

#### 3.1.2. FTIR Analysis

**Figure 3:**
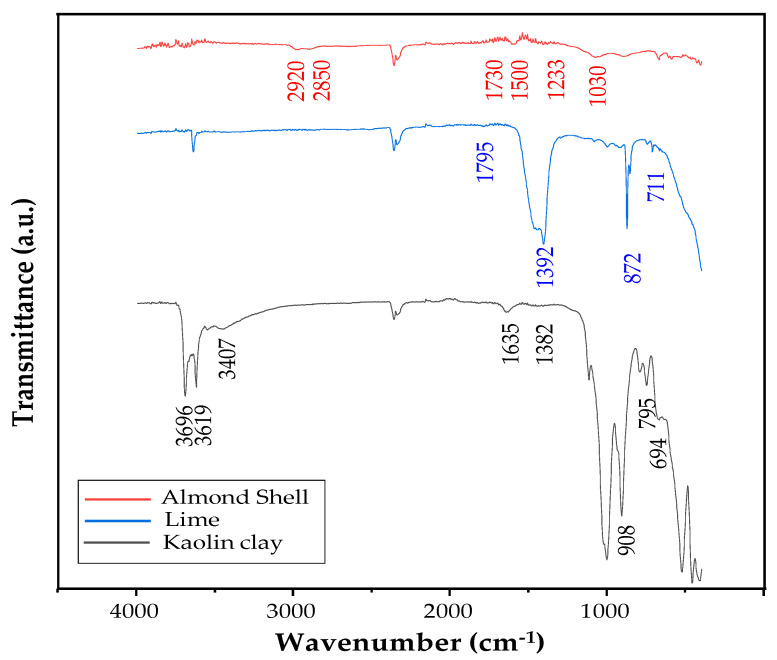
FTIR spectrum from bottom to top of kaolin clay, almond shell and lime.

The IR spectra (Figure 3) identify the functional groups and provide information about their local environment on the surface.

The spectrum of kaolin clay shows bands at 3407, 3619, and 3696 cm^−1^, which can be associated with intermolecular hydrogen bonding between hydroxyl groups linked to Al and Si. The small, broad band observed around 1635 cm^−1^ is assumed to correspond to the symmetrical stretching of the H-O-H bond, related to the water absorbed onto the material [30]. The very weak band at 1382 cm^−1^ can be attributed to the Si-O-Si stretching vibration [41]. On the other hand, the sharp band at 908 cm^−1^ corresponds to the Al-OH group, while the bands at 694 and 795 cm^−1^ are assigned to Si-O-Al bonds [41,42].

The lime spectrum exhibits bands for C-O and C=O vibrations at 1392 and 1795 cm^−1^, respectively, and for Ca-O bond at 872 and 711 cm^−1^ [41].

Although poorly resolved, the characteristic bands of cellulose, hemicellulose, and lignin can be identified in the spectrum of almond shell powder. Lignin produces the more pronounced vibrations: at 2850 and 2920 cm^−1^ corresponding to the -CH stretching of methyl/methylene or methane functionalities, at 1500 cm^−1^ to the stretching C-C and C-O bonds, and at 1233 cm^−1^ to the stretching of C=C in the aromatic ring [40]. In addition, the very weak band at 1730 cm^−1^ is assumed to be due to vibrations of C=O esters in the acetyl group of the hemicellulose component. The band detected around 1030 cm^−1^ is attributed to C-N stretching, which could indicate the presence of primary amines, as reported in a previous work [30]. These chemical signatures explain the reactivity and interactions between components, directly influencing the stability, degradation, and mechanical properties of the membranes. Thus, the FTIR provides fundamental understanding of molecular mechanisms underlying the changes observed in the composite material.

#### 3.1.3. XRD Analysis

**Figure 4:**
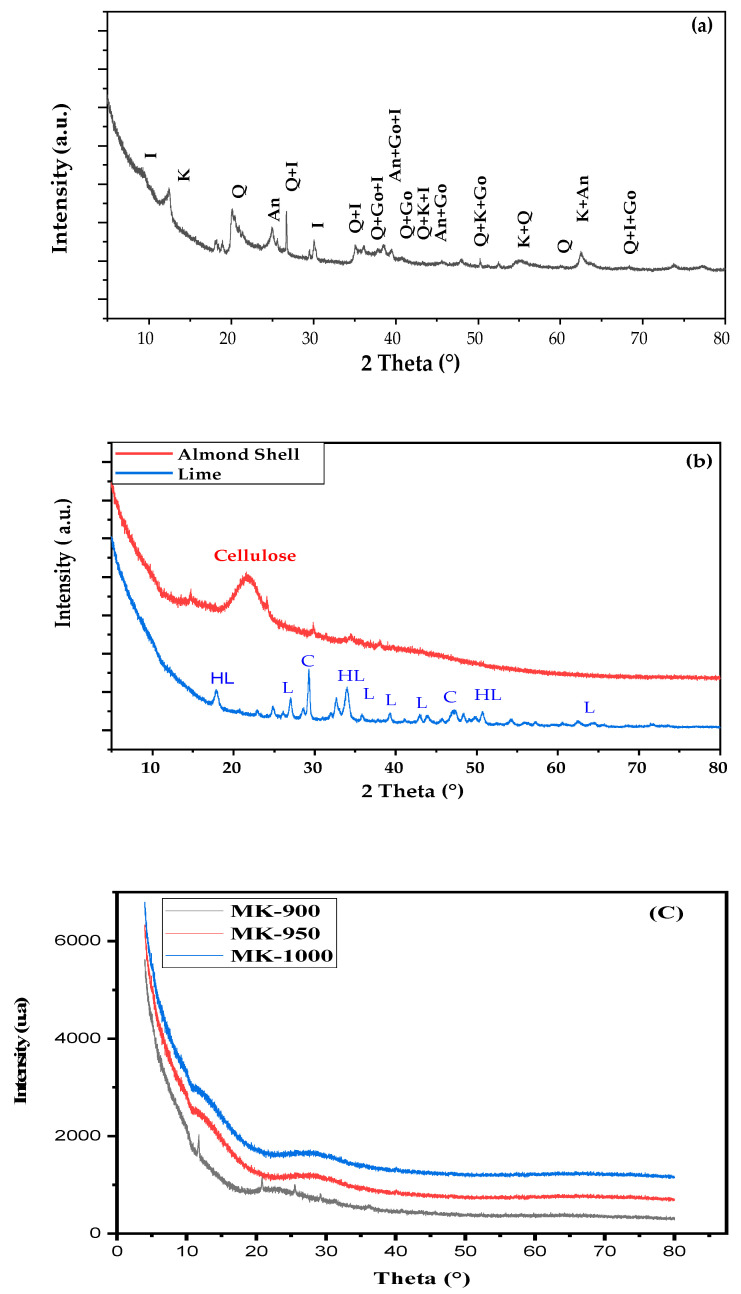
XRD patterns of (**a**) kaolin, (**b**) almond Shell and lime, (**c**) MK.

The crystallinity and composition of the different raw materials were studied by XRD. Figure 4a shows the diffractogram of kaolin clay, and Figure 4b shows those of almond shell and lime used in this study.

The XRD pattern of kaolin clay reveals the presence of quartz (Q), kaolinite (K), illite (I), anatase (An), and goethite (Go) [27,28]. Q and K are known for their low plasticity, high refractory qualities, and hydrophilic properties. However, after sintering, they transform into a liquid phase that serves as a link between the particles, thus improving mechanical characteristics [34]. The XRD study of raw almond shells reveals a peak at around 22°, indicating the presence of specific crystalline phases in the material. This is explained by the fact that lignocellulosic materials exhibit structural defects, which allow the formation of monocrystals known as whiskers [27,28]. This phase is expected to disappear during sintering due to decomposition of cellulose, as previously reported [28]. Figure 4b shows that the XRD pattern of lime is not well defined, as the product used in this study is of technical grade. In addition, two different components besides lime (L) can be identified, due to surface reaction with atmospheric moisture (hydrated lime, HL) and carbon dioxide (calcium carbonate, C) [43,44]. However, lime was regenerated by heating during membrane preparation (see the following section).

Curve (c), obtained by X-ray diffraction (XRD) of the membranes, highlights the progressive decomposition of cellulose, consistent with previous reports [28]. This degradation is particularly evidenced by the disappearance of the characteristic peak at 22°, indicating a significant loss of cellulose crystalline structure within the membranes.

#### 3.1.4. Thermogravimetric Analysis

Thermogravimetric (TGA) analysis aimed to identify the temperature ranges where weight loss and modifications occurred during membrane preparation. Heat treatment can result in the loss of H_2_O, CO_2_, and/or organic matter, as well as a series of mineral structural changes in the raw materials, as previously mentioned.

**Figure 5:**
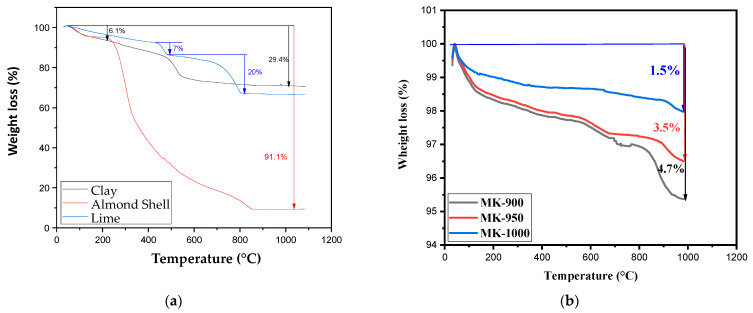
(**a**) TGA curves for kaolin clay, almond shells and lime. (**b**) TGA curves for MK-900, MK-950 and MK-1000.

Figure 5a presents the thermograms from room temperature up to 1000 °C for kaolin clay, almond shell and lime samples. In the case of kaolin, three stages can be observed: firstly, a slight steady decrease up to 500 °C due to the evaporation of adsorbed water (ca. 6%) and kaolinite dehydroxylation; this is followed by a sharp decline after this temperature, corresponding to the polymorphic change from α-quartz to β-quartz; and finally, a stabilization in weight up to 1000 °C with a total loss of around 30%. The thermogram for lime shows a similar profile, with an initial slight decrease corresponding to the loss of adsorbed moisture. This first stage is followed by a dehydroxylation step occurring between 450 and 550 °C, and a decarboxylation between 700 and 800 °C (Equations (11) and (12)) [45].

Dehydroxylation   Ca(OH)_2_→CaO + H_2_O (11)

Decarboxylation   CaCO_3_→CaO + CO_2_ (12)

Calculations based on the respective losses for these two reactions showed that lime was composed of approximately 7% hydrated lime and 20% carbonated lime. Another conclusion from this analysis was that a minimum sintering temperature of 850 °C should be applied. The TGA analysis of the MK membranes indicates that the majority of volatile components decompose before 850 °C, suggesting that beyond this temperature, changes in shrinkage and strength are mainly due to sintering and particle consolidation rather than mass loss. As expected, the almond shell powder is almost completely decomposed in this temperature range by pyrolysis of cellulose and hemicelluloses [30].

Figure 5b shows a minor weight loss after sintering, observed between 25 and 1000 °C, ranging from 1.5% for MK-900 to 4.4% for MK-1000. This loss is mainly related to the removal of hygroscopic water and the evaporation of surface water. The variation in percentage weight loss with sintering temperature can be attributed to the departure of small amounts of organic matter associated with the samples. Therefore, these minor weight losses indicate that the sintered membranes maintain good thermal stability.

### 3.2. Characterizations of the MK Membranes Prepared

#### 3.2.1. Shrinkage and Mechanical Strength

**Figure 6:**
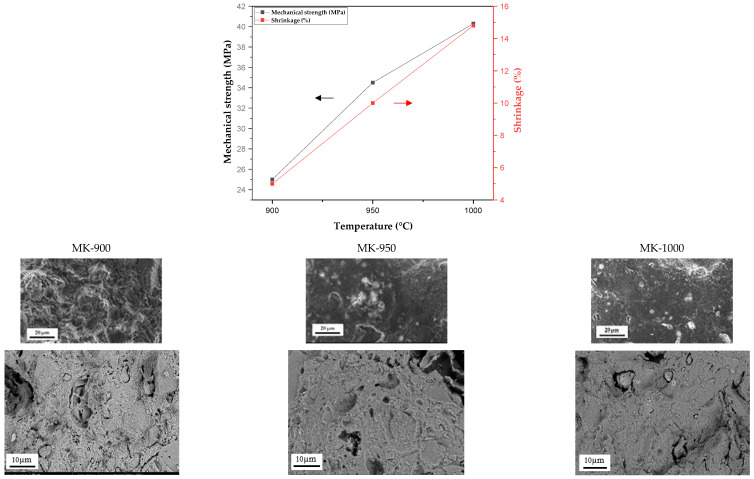
Variation in mechanical strength and shrinkage as a function of sintering temperature for MK-900, MK-950 and MK-1000 membranes, and SEM images of the corresponding membrane surface and cross-section.

Figure 6 illustrates the evolution of shrinkage and mechanical strength of MK-900, MK-950, and MK-1000 membranes produced from slurry composed of 96% kaolin clay, 2% lime and 2% almond shells, as a function of the sintering temperature (900–1000 °C). Shrinkage is often associated with weight loss or particle rearrangement caused by evaporation, combustion, or decomposition during heat treatment. Shrinkage increased almost linearly from 5% to 14.8% when the sintering temperature was raised from 900 to 1000 °C. Similarly, mechanical strength increased, ranging from 25 MPa to 40 MPa over the same temperature range. As revealed by the TGA analysis, the degree of sintering and densification of the materials is the most relevant explanation for the results observed, as no significant evolution can be detected at temperatures higher than 850 °C.

The surface morphology of membranes sintered at different temperatures was studied by SEM. As shown in Figure 6, surface roughness decreases with increasing sintering temperature. Membranes sintered at lower temperatures have a more porous architecture than those sintered at higher temperatures. The surface appears almost uniform in the latter conditions, which confirms the densification hypothesis previously stated to explain the change in mechanical strength.

High temperatures are known to promote the strengthening of intermolecular bonds and improve the overall structural integrity of the kaolinite membrane. This observation is consistent with previous findings reported in the literature [28,34,41,46]. It should be noted that the high percentage of shrinkage at 1000 °C leads to significant membrane deformation. Consequently, only sintering temperatures of 900 °C and 950 °C were taken into account in the study of filtration properties.

**Figure 7:**
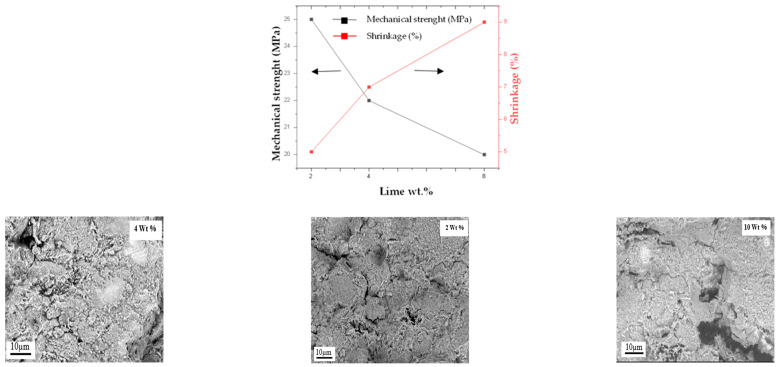
Variation in mechanical strength and shrinkage as a function the lime content (wt.%) for MK-900 membrane sintered at 900 °C and SEM images of the corresponding membrane surface.

Figure 7 shows the evolution of the same parameters for the MK-900 membrane as a function of the amount of lime added (wt.%). An increase in shrinkage from 5% to 9% is observed when the amount of lime increases from 2 to 8 wt.%, probably because it acts as a cohesive binder between the kaolin particles. However, mechanical strength decreases over the same range, from 25 MPa to 20 MPa. Microscopic observation of the membrane surface (Figure 7) shows the formation of morphological defects (pinholes and cracks) beyond 2 wt.%, most likely due to the release of gas associated with the presence of hydrated and carbonated lime (Reactions (11) and (12)). For this reason, the optimal quantity of lime added was set at 2 wt.%. The content of almond shells was also set at 2% to limit the formation of large pores.

#### 3.2.2. Chemical Stability

**Figure 8:**
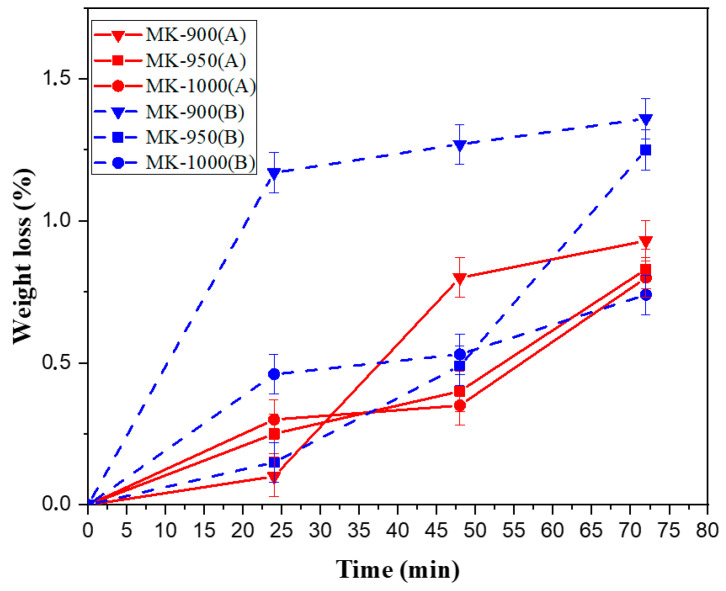
Change over time in the weight loss of MK-900, MK-950 and MK-1000 membranes in acidic (A: in red) and basic (B: in blue) media.

Due to fouling phenomena, cleaning steps are required in membrane separation technology, particularly in the case of the UF process. Therefore, the behavior of ceramic membranes is tested at acid and alkaline pH to check their ability to resist the chemical treatments generally used [28]. To this aim, the MK-900, MK-950 and MK-1000 membranes were tested in acidic and basic media for 72 h at room temperature. Figure 8 shows the variation in weight loss over time when the membrane is immersed in pH = 3 (HNO_3_) and pH = 12 (NaOH). The membrane weight loss is much less than 1.5% after 72 h in all cases. The chemical stability is slightly better in acidic than in basic medium. These results are similar to those reported for other ceramic membranes [34].

#### 3.2.3. Porosity

The porosity of the membranes was calculated using Equation (2), based on the dry and wet weights of the membranes. A slight decrease in the porosity was observed when the sintering temperature increased from 900 to 1000 °C. The calculated porosity values were 29.7%, 27.4%, and 25.6% at sintering temperatures of 900 °C, 950 °C, and 1000 °C, respectively. The densification of the porous structure mentioned above most likely explains the decrease in porosity observed [28,34].

**Figure 9:**
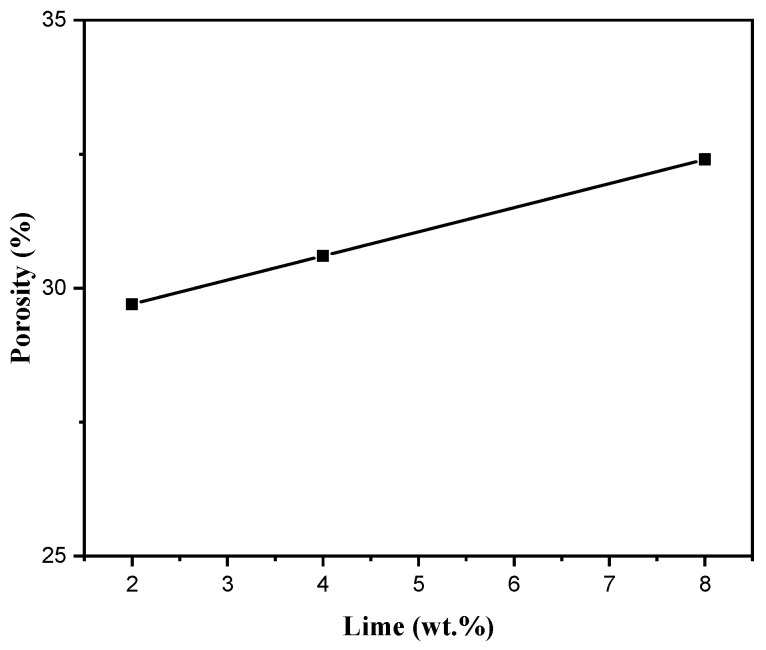
Porosity variation in the MK-900 membranes as function of the lime content in the slurry formulation.

Another important parameter that can affect porosity is the amount of lime added in the slurry formulation. Figure 9 illustrates this effect for the MK-900 membrane. As can be seen, porosity increased from 29.7% to 32.4% when the lime content was increased from 2 to 8 wt.%. This observation can be related to the formation of structural defects demonstrated in Section 3.2.1.

#### 3.2.4. Water Permeability and Pore Size Determination

**Figure 10:**
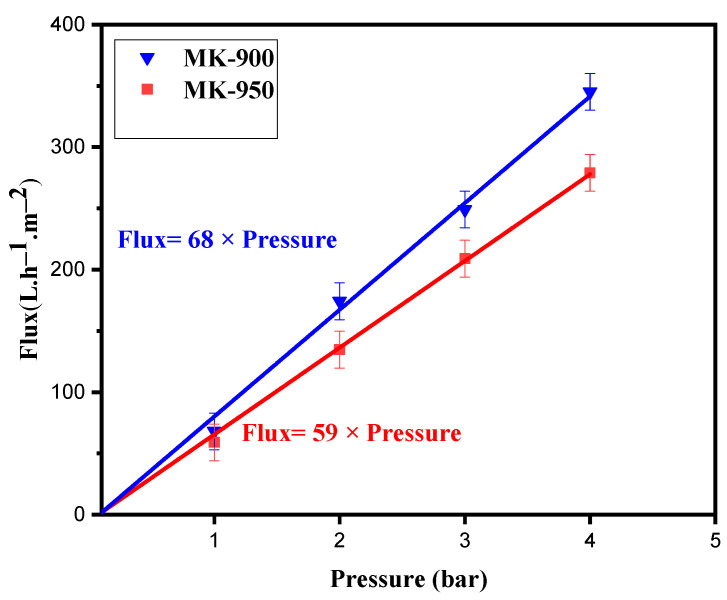
Water permeability of MK-900 and MK-950 membranes.

Water permeability is important in determining the optimal operating conditions for the membrane. MK-900 and MK-950 membranes, prepared from a slurry containing 2 wt.% lime and 2 wt.% almond shells, were selected for the permeation experiments because of their good mechanical stability and porosity. The membranes were soaked in distilled water for 24 h before use to ensure stable permeate flow. Permeability tests were then conducted using pure water at pressures ranging from 1 to 4 bar. Figure 10 shows a linear evolution of the water flux when the pressure increases from 1 to 4 bar. This confirms the verification of Darcy’s law [47], showing the relationship between water permeate flux and the applied transmembrane pressure. The flux increases steadily with pressure in this range and exhibits a linear behavior for the two membranes selected. The water permeability values, determined from the slope of the straight line, were 68 and 59 Lm^−2^h^−1^bar^−1^ for MK-900 and MK-950, respectively. The decrease in permeability with higher sintering temperatures can be attributed to the corresponding densification of membrane structure, along with a decrease in porosity.

The average pore size was estimated using the Hagen-Poiseuille equation (Equation (3)). The calculated values were 44 nm and 42 nm for MK-900 and MK-950, respectively. As expected, the chosen formulation resulted in mesoporous membranes that could be suitable for ultrafiltration applications.

The main results related to the characterization of the different tubular membranes (MK-900, MK-950 and MK-1000) are summarized in Table 2. It is clear that shrinkage, mechanical strength, and average pore size increased with sintering temperature. However, thickness and water permeability decreased.

### 3.3. Application to the Removal of Indigo Blue (IB) Dye

In this section, the MK-900 and MK-950 membranes selected were applied to the treatment of a synthetic textile wastewater containing the IB dye. Its composition was prepared according to the recommendations of the textile company. It was representative of dye baths incorporating auxiliary agents. The main feed parameters, including turbidity, color and chemical oxygen demand, are summarized in Table 3.

**Figure 11:**
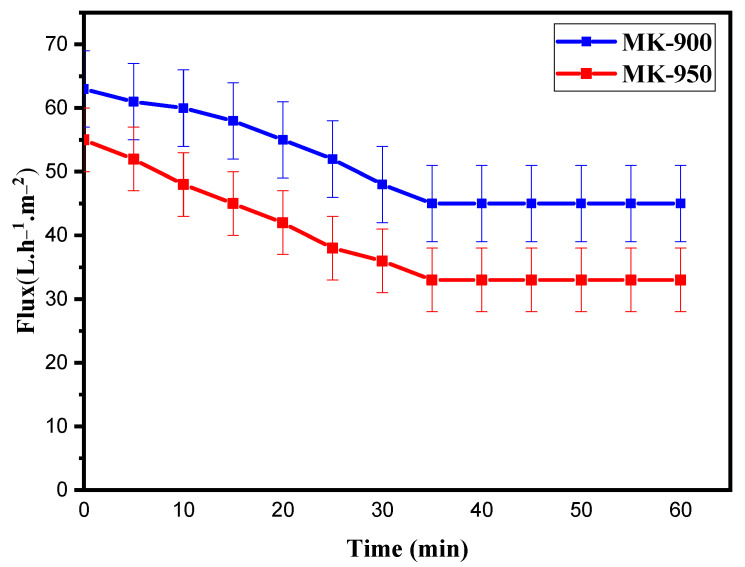
Permeate flux for MK-900 and MK-950 membranes as a function of filtration time (∆P = 1 bar; T = 25 °C).

Figure 11 shows the variation in permeate flux at 1 bar as function of filtration time for both membranes. The flux decreased with time from 64 to 46 Lm^−2^h^−1^ (LMH) for MK-900 and from 55 to 35 LMH for MK-950, reaching a plateau after 35 min for both membranes. The formation of concentration polarization layer and fouling due to the interaction between the solution and the membrane surface is considered to be the cause of the decrease in permeate flux with time [48]. Once the dynamic polarization layer has been established, the permeate flux stabilizes as a function of the operating filtration conditions [49]. This surface layer acts as a new membrane with its own permeation characteristics, inducing additional resistance to the passage of filtrate. It should be noted that the two membranes tested exhibit similar behavior, with a symmetrical evolution off low rate, probably due to the proximity of their pore sizes. Interestingly, the decrease in permeate flux is limited equal to about 37% for both membranes. The flow rate obtained on the plateau is between 35 and 45 LMH at 1 bar, making the prepared MK membranes well suited to wastewater treatment applications.

It appears that the MK-900 and MK-950 membranes effectively removed pollutants from synthetic solutions representative of textile wastewater. Table 3 shows that turbidity decreased dramatically after filtration with almost quantitative retention. On the other hand, both membranes showed rejections for color and COD with similar values. Assuming that IB is mainly responsible of the feed color, this means that COD in the feed is primarily due to the dye molecules. However, the mean pore size of MK membranes is about two orders of magnitude larger than IB molecules and cannot account for the rejection values observed. In fact, IB is only sparingly soluble in water, so it probably forms aggregates that could be retained by the dynamic surface layer. The rough, irregular surface of MK-900 membrane (Figure 6) probably involves the formation of a looser structure of the concentration polarization layer, which could explain the difference in color rejection between the two membranes. Nevertheless, it can be concluded that the permeate showed good discoloration, indicating that the MK membrane can be useful for retaining the color of textile wastewater, especially in the case of MK-950 (R = 92%).

### 3.4. Fouling Study and Regeneration of Membrane Performance

#### 3.4.1. Fouling Study

Membrane fouling by organic or inorganic contaminants is the major problem in the application of membrane technology as it affects permeate flow and separation efficiency. Cleaning steps are then necessary to restore membrane performance, at a frequency and depth that depend on the type of fouling. Therefore, a study was carried out by physically cleaning MK membranes with deionized water after filtration to determine the permeation resistance related to the nature of fouling by using Equations (7)–(10).

The results obtained for the two selected membranes are presented in Table 4. Flux recovery rate (FRR) is a parameter indicative of the antifouling properties of the membrane surface. A value close to 100 means that initial permeation performance can be recovered by simple physical cleaning with water. A decrease in FRR was observed with increasing sintering temperature from 900 °C to 950 °C. Membrane MK-900 showed a very good FRR value close to 90%. This result is consistent with the looser structure of the cake layer formed, as proposed in the previous section to explain the lower color retention of the MK-900 membrane compared to the MK-950 membrane. This structure makes it easier to remove during physical cleaning. This behavior is clearly demonstrated by the calculated values for the rate of reversible (*R_r_*) and irreversible (*R_ir_*) flux decline. In the case of MK-900 membrane, the reversible fouling accounts for about 60% of the total flux decline (*R_t_*) whereas the ratio is only of 50% for MK-950 one. The irreversible fouling in UF membrane is generally considered to be pore clogging requiring thorough and/or chemical cleaning. For this reason, in the following section, a more-in-deep cleaning process involving several backwashes was carried out to examine the behavior of the membranes during successive filtration cycles.

#### 3.4.2. Regeneration of Membrane Performance After Successive Filtration of the Dye Solution

In this section, the MK-900 and MK-950 membranes were cleaned by backwashing with deionized water after filtration of the synthetic textile wastewater. The procedure was repeated three times to ensure optimal removal of foulants. At the end of the cleaning operation, pure water permeability was measured to determine regeneration efficiency [50].

**Figure 12:**
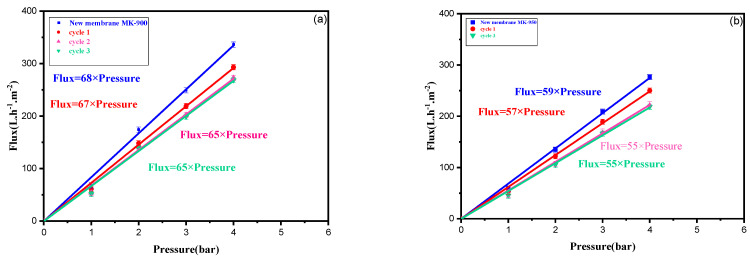
Membranes regeneration after the treatment of the textile wastewater by backwashing with deionized water (**a**) MK-900 and (**b**) MK-950.

Three cycles consisting of filtration of the dye-containing solution followed by regeneration as described above were carried out. The variation in the pure water flux obtained for the two membranes is a linear relationship as a function of applied pressure (Figure 12a,b). From Equation (5), the slope gives the pure water permeability (*L_p_* in Lm^−2^h^−1^bar^−1^**)**. The values obtained are compared with those of the virgin membrane in Table 4 and the rate of change after each cycle is calculated (Table 5).

As can be seen, (Figure 12) thorough cleaning with water appears sufficient to achieve over 90% recovery of the initial permeability for both membranes after three cycles. A similar behavior has been previously reported for a UF membrane made from kaolin clay [34]. This result indicates that fouling due to pore clogging can be almost completely eliminated by backwashing. Moreover, the calculation of the rate change after each cycle showed that the loss of permeability occurred mainly during the first two cycles, as only a very slight decrease was observed between cycles 2 and 3, indicating that a quasi-steady state of fouling had been reached. The irreversible adsorption of hydrophobic and/or positively charged molecules on the kaolinite surface could explain this observation. The residual loss of permeability would come from the resulting restriction of pores. It should be noted that adsorption would stop once the pore surface is covered. Furthermore, the smallest pores, which are more difficult to clean, are probably the most affected by this phenomenon.

#### 3.4.3. Cost Analysis

Membrane production costs were evaluated based on raw material expenses and energy consumption. Conventional ceramic membranes, typically fabricated from high-purity metallic oxides (such as alumina, zirconia…), incur significant expenses ranging from $500–1000/m^2^ [18] due to valuable precursors and intensive sintering temperatures. Table 6 details the cost breakdown for a kaolin-based membrane, including:

- Raw material procurement and modification,
- Shaping processes,
- Sintering energy requirements.

The developed sustainable ceramic membrane demonstrates exceptional cost efficiency at $15.97/m^2^, establishing a competitive advantage in ultrafiltration wastewater treatment. Comparative analysis reveals significant savings across key benchmarks:

- 9% reduction versus zeolite/smectite ceramic membranes ($17.56/m^2^) [50].
- 20–23.6% savings relative to kaolin/zeolite composites ($20.92/m^2^) [51] and surface-modified zeolite systems ($20.11/m^2^) [52].
- >85% cost reduction compared to agricultural waste-derived alternatives (e.g., walnut shell/kaolin membranes > $112/m^2^) [28].

By maintaining uncompromised separation performance while achieving these economic benefits, the membrane demonstrates both technical viability and superior cost-effectiveness versus existing natural material-based ceramic membranes.

## 4. Conclusions

In this study, it was demonstrated that low-cost, eco-friendly, and sustainable ceramic membranes can be successfully prepared from kaolin powder in a single step, without the need for a membrane support. The combination of lime as a binder and almond shells as a pore-forming agent at a relatively low percentage produced tubular membranes with good physical and chemical properties and an average pore size in the ultrafiltration range, which was the aim of this work. Interestingly, almond shells, an agricultural by-product, have an effective pore forming action, providing good porosity and high membrane permeability.

The membranes prepared with a formulation containing 2 wt.% lime and 2 wt.% almond shells and firing temperatures of 900 and 950 °C showed very good performance for the treatment of textile wastewater using a reconstituted effluent containing IB in terms of discoloration, turbidity and COD removal. Color removal ranged from 78 to 92%, depending on preparation conditions. These results were explained by the formation of a dynamic surface layer acting as an additional top surface filter in the nanofiltration range, enabling high rejection of IB molecules. Interestingly, backwashing the membranes with water restored the initial permeability to over 90% by almost completely removing the top layer. Textile wastewater treatment requires nanofiltration or reverse osmosis to remove color and COD. The prepared MK membranes, which are low-cost, green and sustainable, could be a particularly promising alternative for removing most of the color and COD before further treatment by reverse osmosis, if required.

## Figures and Tables

**Table 1:** Chemical composition (wt%) of the kaolinite clay used in this work.

SiO_2_	Al_2_O_3_	K_2_O	CaO	Fe_2_O_3_	MgO	TiO_2_	LOI ^1^
42.96	37.70	0.94	0.74	0.32	0.23	0.03	16.50

^1^ LOI: Loss on ignition at 1000 °C.

**Table 2:** Main characterization of the synthesized membranes sintered at different temperatures.

T_sintering_ (°C)	900	950	1000
Shrinkage (%)	5	10	15
Mechanical strength (MPa)	25	35	40
Thickness (mm)	4.2	4.0	3.6
Porosity (%)	30	27	26
Water permeability (Lh^−1^m^−2^)	68	59	-
Average pore size (nm)	42	44	-

**Table 3:** Parameters of the synthetic feed solution representative of the IB dye baths (pH 8.5) and of the permeate after filtration with MK-900 and M-950 membranes.

		Turbidity (NTU)	R (%)	Color (Abs^610^)	R (%)	COD (mg/L)	R (%)
Feed		187	-	1.132	-	147	-
Permeate	MK-900	4	98	0.248	78	35	76
	MK-950	2	99	0.023	92	15	90

**Table 4:** Fouling study of the selected MK-900 and MK-950 membranes using Equations (7)–(10).

Membrane	*J_w_*_1_ (Lm^−2^h^−1^)	*J_wf_* (Lm^−2^h^−1^)	*J_w_*_2_ (Lm^−2^h^−1^)	FRR (%)	*R_t_* (%)	*R_r_* (%)	*R_ir_* (%)
MK-900	68	46	59	86	32	19	13
MK-950	59	33	46	79	44	22	22

**Table 5:** Pure water permeability *L_p_* in L m^_2^ h^_1^ bar^−1^ after each regeneration cycle and rate of change from the previous value.

*L_p_* (L m^−2^ h^−1^ bar^−1^)	Virgin	After Cycle 1	Change (%)	After Cycle 2	Change (%)	After Cycle 3	Change (%)	Total Change (%)
MK-900	68	67	1.5	65	3.0	65	<0.5	<5
MK-950	59	57	3.3	55	3.6	55	<0.5	<7.5

**Table 6:** Cost Analysis for the fabrication of the new sustainable membrane.

Price of Raw Materials			
Material	Unit per Kg ($)	Amount of Raw Material (g)	Price ($)
kaolin powder	0.16	384	0.0614
Lime	1	8	0.008
Distilled water	0.28	200	0.056
Almond shells	-	8	-
Total raw materials cost for the fabrication of 15 membranes			0.1254
Energy cost (Based on the power consumption)			
Mixer			0.031
Dry oven			0.027
Extruder			0.138
Furnace			0.086
Total production cost for the fabrication of 15 membranes ($)			0.4074
(Surface of membrane = 1.7 × 10^−3^ m^2^)			
Total production cost of the (MK) Kaolin membrane ($m^−2^)			15.976

## Data Availability

Dataset available on request from the corresponding authors.
